# Contrasted Ethnobotanical and Literature Knowledge of Anti-Mosquito Plants from Guadeloupe

**DOI:** 10.3390/biology14070888

**Published:** 2025-07-19

**Authors:** Yolène Duchaudé, Laura Brelle, Muriel Sylvestre, Anubis Vega-Rúa, Gerardo Cebrián-Torrejón

**Affiliations:** 1 COVACHIM-M2E Laboratory, University of Antilles, BP 250, 97157 Pointe à Pitre Cedex, France; 2 Vector-Borne Diseases Laboratory, Institut Pasteur de la Guadeloupe-Lieu-Dit Morne Jolivière, 97139 Les Abymes, France

**Keywords:** ethnobotany, traditional knowledge, vector control plants, Guadeloupe

## Abstract

The *Aedes aegypti* mosquito, vector of dengue, is a major public health threat in the Caribbean. In Guadeloupe, where dengue outbreaks occur frequently, traditional plant-based remedies are part of the local heritage but remain poorly documented. This study aimed to evaluate the anti-mosquito potential of 38 Guadeloupean plants through an ethnobotanical survey. A semi-structured online questionnaire was conducted over five months, targeting the plant knowledge of residents. Inclusion/exclusion criteria were applied to identify and validate relevant species. Ethnobotanical indices such as Frequency of Citation (FC), Fidelity Level (FL), and Relative Frequency of Citation (RFC) were calculated. Out of the 38 surveyed plants, 22 were confirmed for their traditional anti-mosquito uses. The most cited species included *Cymbopogon citratus* (93.3%), *Artocarpus altilis* (25%), and *Pimenta racemosa* (18.3%). Comparative analysis with existing literature showed that 12 of these plants had not been previously reported for vector control. This highlights the value of ethnobotanical approaches for discovering alternative, eco-friendly vector control options and the importance of preserving traditional knowledge. The study reveals both the high potential of Guadeloupean flora and the risk of cultural erosion, supporting further research into the bioactive compounds of the most cited species.

## Simple Summary

Mosquito-borne diseases are a major public health issue, especially in tropical regions like the Caribbean. In Guadeloupe, traditional knowledge about plants has been passed down through generations but remains poorly documented today. This study aimed to record and analyze the medicinal plants used by the local population to fight mosquitoes through a digital survey involving 216 local informants. Based on interviews, several species reputed for their repellent or insecticidal properties were identified from a list of 38 surveyed plants. Our findings provide valuable insights for 22 plants that were confirmed by respondents for their traditional use against mosquitoes. We also identified 12 species that have not previously been reported in the scientific literature for mosquito control. This study highlights the rich but declining traditional knowledge in Guadeloupe and underscores the potential of its flora for developing natural, eco-friendly solutions to help reduce the spread of mosquito-borne diseases.

## 1. Introduction

Arboviral diseases, including Chikungunya, Dengue, and Zika, transmitted by the infected female vector *Aedes aegypti*, constitute a major public health concern worldwide [1,2,3]. Among these, Dengue, caused by dengue virus (DENV), is currently endemic in tropical and subtropical regions, thus exposing all inhabitants of Africa, Europe, Oceania, and the Americas [2,4]. Dengue symptoms are often mild and vary from patient to patient, but more severe outcomes (i.e., haemorrhagic fever) can also occur and lead to death [5]. In 2023, reported cases of Dengue fever reached an all-time high value, with more than 6.5 million cases and more than 7300 dengue-related deaths [6]. In the Caribbean, and particularly in the Guadeloupe archipelago, epidemics are regularly recorded every 2–3 years [7]. According to several forecasting models, this scenario can worsen as climate change will impact vector-borne disease transmission by promoting the proliferation of breeding sites, blood meals, and viral spread in certain settings [8,9].

Today, we lack long-term solutions to cope with the burden of dengue. As recommended by the Pan American Health Organization [10], various measures have been taken to reduce its impact, including vaccine formulations and the use of chemical insecticides. Regarding dengue vaccines, several options exist, but none cover the entire population, and their efficacy can be influenced by patients’ dengue history. For instance, Qdenga and Dengvaxia vaccines have been reported to have an efficacy of 80% [11], but the latter is only recommended for individuals with prior dengue exposure [12,13,14]. Regarding insecticides, their intensive use impacts the environment and leads to selection of insecticide resistance, as observed in many *Ae. aegypti* populations [15,16,17,18]. Both strategies have so far failed to efficiently avoid DENV spread in the populations, which underlines the urgency and necessity of research on alternative approaches or products for mosquito control.

The traditional use of herbal formulations has already shown effectiveness in repelling or killing mosquitoes in dengue-endemic areas [19,20,21]. Numerous ethnobotanical studies have demonstrated that medicinal plants can be effective due to their active compounds (e.g., essential oils) against mosquitoes [22,23]. Because the population from dengue endemic areas possesses a strong know-how on mosquitoes and their link with the environment, many studies have used ethnobotanical approaches to identify vector control plants (VCP). Through these studies, numerous new pharmaceuticals were developed, and several active ingredients were discovered around the world [20,24,25,26].

Here, to find new ecologically and environmentally friendly solutions for mosquito control, we used an ethnobotanical survey to investigate the ancestral knowledge of Guadeloupe inhabitants on VCPs. Guadeloupe is considered a biodiversity hotspot [27], where several plants are recognised for their culinary, cosmetic, and medicinal properties thanks to previous ethnobotanical and ethnopharmacological studies [22,28]. However, the documentation of Guadeloupean knowledge of VCP is scarce. Building on the TRAMIL guidelines (Program of Applied Research on Popular Medicine in the Caribbean) and previous studies, we aimed to investigate the traditional uses of Guadeloupean plants specifically in the context of vector control [22,23].

In this study, we used an inclusion/exclusion method to identify 38 plants with documented traditional uses and strong potential for mosquito control. The selection process was guided by a multidisciplinary local team comprising botanists, doctors, biologists, social workers, chemists, and local experts. Various criteria were considered, including information from local literature and the presence of volatile organic compounds (VOCs) or essential oils in the plants (Table 1). This collaborative approach ensured a comprehensive evaluation of each plant’s suitability for further investigation based on both scientific and traditional knowledge. Then, we conducted a vector control potential classification based on a digital semi-structured survey conducted in Guadeloupe of those 38 know-how plants.

## 2. Materials and Methods

### 2.1. Description of the Study Area

**Figure 1:**
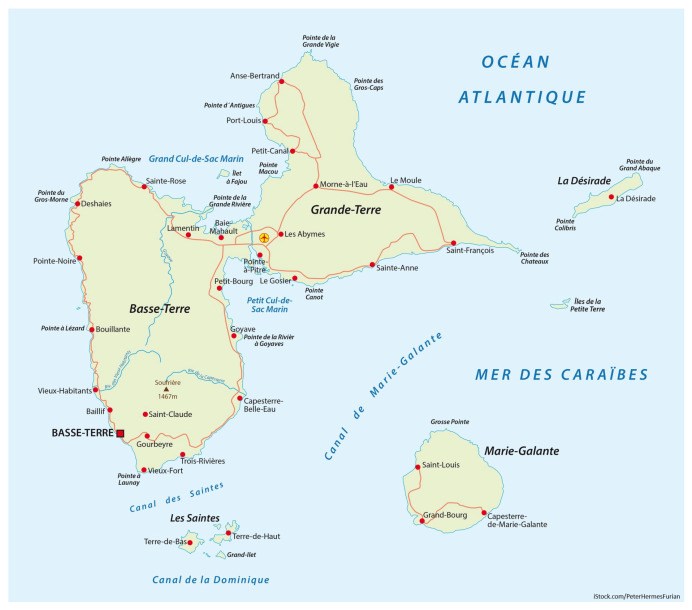
Map of the study area.

Our study area focused on plants recorded in the Guadeloupe archipelago (French West Indies). According to Encyclopædia Universalis France [29], Guadeloupe is located in the Caribbean Sea at 14°47′53″ N 58°24′59″ W and the Map of Guadeloupe archipelago are available in the Figure 1. This partly volcanic island is the largest of the Leeward Islands. Several studies have highlighted Guadeloupe as a biodiversity hotspot [27].

### 2.2. Know-How Plants Selection via Inclusion/Exclusion Method

A list of 38 know-how plants was elaborated by a local multidisciplinary group including botanists, doctors, biologists, social workers, chemists, and local experts. Numerous criteria were considered, including the scientific expertise of the multidisciplinary group, reports from local literature reports [23] and the presence of odorous molecules in the plants. The scientific names, reported uses, and phytochemical compounds of each proposed plant are available in Table 1.

### 2.3. Ethnobotanical Data Collection and Statistical Analysis

In response to COVID-19 restrictions mandated by the Prime Minister [30], we developed a digital semi-structured questionnaire using Google Forms. This online survey was distributed via a snowball method between September 2022 and January 2023, and 216 volunteers over the age of 18 chose to participate after being informed of the scientific purpose of the survey according to procedures of the internal ethics committee of COVACHIM-M2E Laboratory (University of the West Indies).

**Figure 2:**
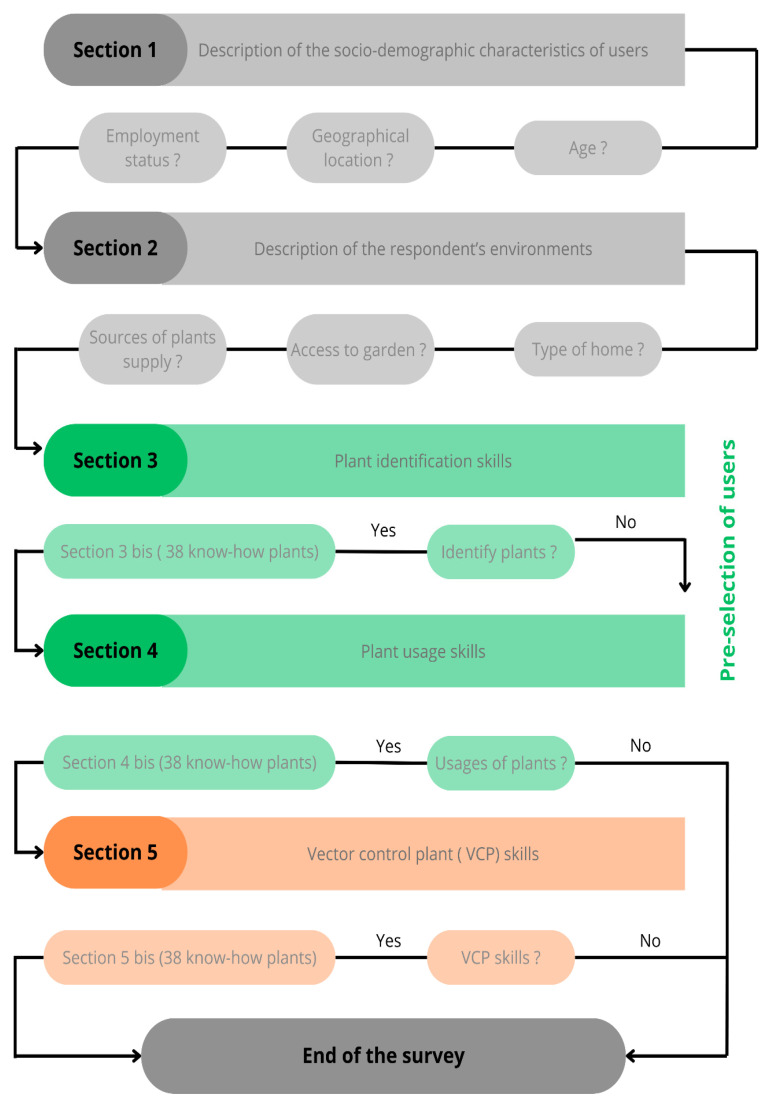
Flowchart of the ethnobotanical survey methodology for assessing plant-based vector control knowledge in Guadeloupe. The survey consists of five main sections: (i) socio-demographic characteristics of participants (grey, Section 1), (ii) description of their environmental context (gray, Section 2), (iii) assessment of plant identification skills (green, Section 3), (iv) evaluation of plant usage knowledge (green, Section 4), and (v) assessment of knowledge on vector control plants (VCP) (orange, Section 5). The “bis” sections (lighter shades) provide an in-depth assessment of participants’ knowledge of 38 documented plants. Arrows indicate the flow of the questionnaire based on participants’ responses (Yes/No), with a pre-selection step ensuring that only participants with recognized plant knowledge proceed further in the survey.

The survey was set up following the TRAMIL methodology, adapted with a few modifications to (I) take into account respondents’ digital fluency, and to (II) pre-select local populations with plant knowledge (III) to assess the plants with potential for vector control properties [22]. Thus, the survey was divided into four sections. The first section provided a description of the socio-demographic characteristics of the respondents: age, geographical location, and employment status. The second section was based on the respondents’ environment: their type of home, their access to gardens, and their source of plant supply. The third section assessed the plant identification skills of respondents, and the fourth section assessed their plant usage skills. The latter two sections can be considered as a pre-selection of users based on their level of ancestral and folklore knowledge related to plant-based practices. A list of 38 know-how plants was presented only to pre-selected users. Thus, uninitiated respondents who could neither identify plants (Section 3) nor use them (Section 4) did not have access to this list. Only respondents with plant knowledge were authorized to select the plants that they could identify in Section 3 bis and the plants they used in Section 4 bis. The Flowchart of the ethnobotanical survey methodology for assessing plant-based vector control knowledge in Guadeloupe is available in Figure 2. The survey questionnaire and the associated sections are available in the Supplementary Materials.

The fifth section classified the users with skills related to vector control, while participants without those skills were redirected to the end of the survey. In Section 5 bis, the 38 know-how plants list was presented to assess the vector control folklore knowledge of Guadeloupean pre-selected respondents. To avoid bias, all the 38 know-how plant names were given with their scientific names and their local names. The Statistical analysis was carried out using GraphPad Prism 9.

### 2.4. Quantitative Etnobotanics

To assess quantitatively the importance of the selected plants in traditional practices, three ethnobotanical indices were considered: Fidelity Level (FL), Frequency of Citation Index (FC), and Relative Frequency of Citation Index (RFC). These indices provide complementary insights into the relationship between plant selection and their reported uses by respondents.

#### 2.4.1. Fidelity Level (FL)

This formula was calculated as described in Friedman et al. [24]. In this adapted formula, IP represents the number of respondents who selected a particular plant species for a specific use, and IU represents the total number of respondents who selected the same plant across the entire survey. This metric provides insight into the relationship between a selected plant and its reported use by users.

$$FL=\frac{IP}{IU}\times 100$$

#### 2.4.2. Frequency of Citation Index (FC)

The citation frequency (FC) is described as a percentage (%) and was adapted using the formula in Fanou et al. [31].

$$FC=\frac{Ns}{Nt}\times 100$$

where Ns is the number of times a plant was selected in one section and Nt is the total number of all selected species in the same section.

#### 2.4.3. Relative Frequency of Citation Index (RFC)

This formula, adapted from [32], was calculated from the citation frequency (FC), where FC is the frequency of a selected plant in relation to the total number of plants selected per section, and N is the total number of respondents per section.

$$RFC=\frac{FC}{N}$$

### 2.5. Previous Knowledge of Plants’ Anti-Mosquito Properties

To assess the general knowledge about the 38 know-how plants proposed and to confirm their vector control potential when information was available, a literature review was conducted through a process of inclusion/exclusion of keywords for each plant. As a first step, two databases were selected: Science Direct and PubMed (National Library of Medicine). To search for articles, we used each scientific plant name combined with height different keyword in these databases. Articles suggested by the databases were selected for initial abstracts and keywords screening. We removed from our selection irrelevant abstracts and keywords, duplicates, non-open-access articles, and articles published before 2010 before validation of the final articles list that was included in the analysis.

## 3. Results

### 3.1. Preselection of 38 Candidate Plants by a Multidisciplinary Group

Drawing on the diverse expertise of a multidisciplinary group and literature data available, a targeted list of plant species with promising insecticidal or repellent potential was established. This collaborative work led to the identification of 38 candidate species. Table 1 provides an overview of these 38 preselected species, including their reported traditional uses, major phytochemical constituents, and relevant literature references. Insecticidal properties, when documented, are highlighted in bold. The selected species are distributed across 24 botanical families, with Lamiaceae, Fabaceae, Rutaceae, and Zingiberaceae being the most frequently represented. These families are known for their rich essential oil profiles and bioactive compounds such as terpenes, flavonoids, alkaloids, and phenolic acids.

Some species, such as *Azadirachta indica*, *Ocimum basilicum*, and *Cymbopogon citratus*, have already been studied in the context of mosquito control. Others, although less documented, exhibit phytochemical profiles suggesting potential bioactivity. This scientifically grounded selection served as a basis for the ethnobotanical survey.

**Table 1:** Overview of the 38 know-how plants: traditional uses, properties, and phytochemical composition. Insecticidal properties documented were underlined and bolded.

Scientifics Names Plants (Family)	Reported Tradional Uses	Phytochemical Composition (Main Compound)	References
*Aloe barbadensis* Mill. (Asphodelaceae)	Constipation, liver and skin disorders, conjunctivitis, headache, diabetes treatment	Anthraquinones, flavonoids, tannins, sterols, alkaloids, and VOCs.	[23,33]
*Alpinia zerumbet* (Pers.) B.L.Burtt & R.M.Sm. (Zingiberaceae)	Flu-like symptoms, digestion, gastric ulcer, high blood pressure	Terpenes, essential oils, flavonoids, polyphenolics, and sterols	[23,34]
*Anethum graveolens* L. (Apiaceae)	Digestion, infant and adult colic, helps breastfeeding	Flavonoids, proteins, lipids, glucides, fibers	[23,35]
*Annona muricata* L. (Annonaceae)	Anti-tumor, anti-helminth, anti-fungal, anti-bacterial, hypotensive, anti-viral, and anti-inflammatory effects	Phenolic compounds, acetogenins, and alkaloids	[34,36,37,38,39,40]
*Annona squamosa* L. (Annonaceae)	Skin rash, cardiotonic, digestion, flu, **insecticidal**	Flavonoids, phenolic compounds, quinones, coumarins, amino acids, anthocyanidins, and sugars	[23,36]
*Artocarpus altilis* (Parkinson) Fosberg (Moraceae)	Liver disorder, adjuvant to hypertensive treatments	Phenolic compounds	[23]
*Azadirachta indica* A. Juss. (Meliaceae)	Pyrexia, headache, ulcer, respiratory disorders, cancer, diabetes, leprosy, malaria, dengue, chicken pox, and dermal complications	Phenols, tannins, leucoanthocyanidins, catechins, favonols, and xanthones	[23,41]
*Bixa orellana* L. (Bixaceae)	Fatigue, sunburn, diarrhea	Phenols, alkaloids, and flavonoids	[23,42]
*Carica papaya* L. (Caricaceae)	Liver disorder, worms, abscess, boil, digestion, ulcerative wounds, urethritis	Lipids, sulfur compounds, benzenoids, phenolic compounds, proteins, vitamins, alkaloids, carotenoids, and tannins,	[23,36]
*Chrysopogon zizanioides* (L.) Roberty (Poaceae)	**Insect repellent,** deodorant, gastrointestinal colic, nervousness	Terpenes	[23,43]
*Citrus* × *aurantiifolia* (Christm.) Swingle (Rutaceae)	Flu condition, colds, cough, sore throat, gingivitis, digestion, arteriosclerosis, venotomy, liver disorder, rheumatism, **insect repellent,** wounds, cosmetics, conjuctivitis	Flavonoids, terpenes, phenolic compounds, limonoids, alkaloids, and essential oils	[23,44]
*Coleus amboinicus Lour.* (Lamiaceae)	Difficult digestion, wounds and **insect bites**, painful periods, nervousness	Terpenes, phenolic compounds, flavonoids, esters, alcohols, and aldehydes.	[23,45]
*Cucumis anguria* L. (*Cucurbitaceae*)	Treat stomach pain and to reduce oedema, treat jaundice, urolithiasis (formation of kidney stones)	Alkaloids, flavonoids, tannins, carotenoids, steroids, and anthocyanins,	[23,46]
*Curcuma longa* L. (Zingiberaceae)	Anticancer, antidiabetic, anti-osteoarthritis, antidiarrheal, cardioprotective, anti-oxidant, neuroprotective, hepatoprotective, anti-microbial, renoprotective and anti-inflammatory activities	Phenolic compounds, terpenes, phytosterols, and essential oils	[23,47]
*Cymbopogon citratus* (DC.) Stapf (Poaceae)	Digestion, **insect repellent**	Terpenes, phenylpropanoids, phenolic acids, esters, flavonoids, flavone, fatty alcohol and phytosterols	[23,48]
*Dianthera pectoralis* (Jacq.) J.F.Gmel. (Acanthaceae)	Cough, gastrointestinal colic, superficial wounds, nervousness, boils, insomnia	Alkaloids, flavonoids, steroids, terpenes, saponosides, and phenolic compounds	[23,36]
*Elymus repens* (L.) Gould (Poaceae)	Urolithiasis and urinary tract infections, improve the microcirculation, improve body’s defense, mechanisms, activate contractions of uterus, heal atherosclerosis, treat wounds and promote the differentiation and trigger the division of keratinocytes in humans	Flavonoid glycosides and sterols	[23,49]
*Eryngium foetidum* L. (Apiaceae)	Antibacterial, antiviral, and antipyretic applications	Aromatic and aliphatic aldehydes, carotenoids, flavonoids, phenolic compounds	[23,35]
*Euphorbia hirta* L. (Euphorbiaceae)	Measles, inguinal lymph node disease, diarrhea	Triterpenes, flavonoids, xanthones, and polyphenols	[23,36]
*Hibiscus* × *rosa-sinensis* L. (Malvaceae)	High blood pressure, prevention of urinary disorders, cough, conjunctivitis, headaches	Flavonoids, lipids, alcanes, terpenes, carboxylic acid, proteins, glucids, and minerals	[23,36]
*Laportea aestuans* (L.) Chew (Urticaceae)	Heartburn, nausea, dyspepsia, vomiting, flatulence, reflux, ulcer, restlessness, decreased appetite	Steroids, tannins, phenols, flavonoids, and alkaloids	[50,51]
*Lippia alba* (Mill.) N.E.Br. ex Britton & P.Wilson (Verbenaceae)	Flu condition, difficult digestion, gastrointestinal ulcer	cyclic ether, alcohols, monoterpenes, sesquiterpenes, and ketones	[23,52]
*Malpighia emarginata* DC. (Malpighiaceae)	Treatment of symptoms related to respiratory, cardiovascular and cholesterol-related diseases	Saccharides, amino acids and vitamins	[23,53]
*Mangifera indica* L. (Anacardiaceae)	Diarrhea, water retention, respiratory tract conditions, rheumatism, herpes	polyphenolic acids, benzophenones, flavonoids, ascorbic acid, carotenoids, and tocopherols	[23,46,54]
*Mimosa pudica* L. (Fabaceae)	Menstrual cramps	Sesquiterpenes, tannins, and proteins,	[23,36]
*Mirabilis jalapa* L. (Nyctaginaceae)	Boils, sprain, contusion	Glucids, steroids, alcanes, alcohols, cetones, triterpenes, flavonoids, saponins, and iridoids	[23,36]
*Momordica charantia* L. (Cucurbitaceae)	Rash, Flu condition, **insecticidal**, plant protection, superficial skin disorder, pediculosis	Alkaloids, phenolic compounds, flavonoids, saponosids, steroids, terpenoids, tannins, triterpenes, amino acids, glucids, saponins, carotenoids	[23,36]
*Moringa oleifera* Lam. (Moringaceae)	Burns, anti-inflammatory, antinociceptive, antiatherosclerotic, oxidative DNA damage protective, antiperoxidative, cardioprotective	Phenolic acids, flavonoids, alkaloids, phytosterols, natural sugars, vitamins, minerals, and organic acids	[23,55]
*Neurolaena lobata* (L.) *R. Br. ex Cass.* (Asteraceae)	**Malaria**, flu, fever, blood detoxification, diabetes and heal wounds and infections	Saponins, tannins, alkaloids, and flavonoids	[23,56]
*Ocimum basilicum* L. (Lamiaceae)	Difficult digestion, headache, vertigo, joint pain, common cold, sinusitis, skin rash, **insect bites**	Terpenes, alkaloids, flavonoids, tannins, saponins, glycosides, ascorbic acid	[23,52,53]
*Phyllanthus amarus Schumach. & Thonn.* (Phyllanthaceae)	Digestive disease, jaundice, renal calculus	Carbohydrates, triterpenoids, alkaloids, glycosides, tannins, flavonoids, polyphenols, triterpenes, and sterols	[23,57]
*Pimenta racemosa* (Mill.) J. W. Moore (Myrtaceae)	Rheumatism, bruises, Flu condition, tooth pain, headaches	Phenylpropanoids, monoterpenes, phenolic compounds, and terpenes	[23,36]
*Psidium guajava* L. (Myrtaceae)	Diarrhea, superficial skin disorder, nervousness, vomiting, hangover	Flavonoids, triterpenes, benzenoids, thiazoles, sulfur compounds, thiophenes, steroids, lipids, coumarins, alKanes, alKenes, and oxygenated compounds	[23,36]
*Senna alata* (L.) Roxb. (Fabaceae)	Antiallergic, anti-inflammatory, antioxidant, anticancer, antidiabetic, and antifungal	flavones, flavonols, flavonoids, glycosides, anthraquinones and sterols	[23,36]
*Sphagneticola trilobata* (L.) Pruski (Asteraceae)	Painful periods, bronchitis, vomiting	Sesquiterpenes, diterpenes, and triterpenes	[23,36]
*Tetradenia riparia (Hochst.) Codd* (Lamiaceae)	Treat respiratory problems, cough, headache, stomach pain, diarrhea, fever, **Malaria and Dengue**	Pyrone, diterpenes, terpenes, and essential oils	[58]
*Zanthoxylum caribaeum Gaertn.* (Rutaceae)	Acaricidal, antimicrobial, antioxidant, and **insecticidal properties**	Steroids, flavonols, flavones, flavononols, tannins, triterpenoids, and xanthones	[36,59,60]
*Zingiber officinale Roscoe* (Zingiberaceae)	Digestive conditions, motion sickness dizziness, oropharyngeal conditions, dental pain, tonic, wounds, Flu condition, cough, cholesterol, prevention of atherosclerosis, rheumatism	Phenolic compounds, terpenes, polysaccharides, lipids, organic acids, and raw fibers	[61]

### 3.2. Sociodemographic Characteristics of Responders

A total of 216 people were interviewed in the survey. Among them, 62.5% were women (135/216), 22.2% were men (48/216), and 15.3% did not provide any details. Participants were asked about their field of work and their age. Only 3.7% (8/216) of the participants worked in the agricultural field, and the main age group comprised participants between 36 and 50 years old, representing 6.9% (2/29) of users. Among the participants, (20.4% or 44/216) worked in the health sector, with the largest age group being 27.4% (29/216) for those aged between 18 and 35 years old. Similarly, 13.4% (29/216) of participants worked in research, with the main age group being 24.1% (7/29) for those aged between 36 and 50 years old. Additionally, 12% (26/216) of participants were employed in education, where the highest age group was 32% (16/50), for those aged between 51 and 65 years old. Half of the participants (50.5% or 109/216) work in another field not proposed in this study, and the most common age group of these participants was between 18 and 35 years old. All the information regarding work sectors by age group is available in Table 2. Almost all the participants (98.1%) lived in Guadeloupe or the Caribbean basin area, as expected; other locations were not surveyed.

Finally, 76.9% (166/216) of participants had access to a garden, while 23.1% (50/216) of them did not. Of users with garden access, they revealed that 10.8% (18/166) of them lived in an apartment and 89.2% (148/166) of users lived in a house. Regarding users without access to a garden, 70% (35/50) of them lived in apartments, while 30% (15/50) lived in a house.

### 3.3. Pre-Selection of Participants Based on Plant Knowledge and Usage

A pre-selection method was developed to distinguish between users, those with no plant identification skills (I), those who utilize plants (II), and those who recognize plants’ vector control properties (III). According to our pre-selection method, 6% of total participants (13/216) were unable to identify Caribbean plants in Section 3. The remaining participants (94% or 203/216) were asked to select the plants that they could identify in Section 3 bis. In addition, because they could not list their use of Caribbean plants in Section 4, 13.4% (29/216) of them were redirected to the end of the survey. Thus, a total of 86.6% (187/216) of participants were assessed on their general plant usage in Section 4 bis. However, among them, only 32.1% (60/187) declared using plants also for their vector control properties in Section 5. These participants were then asked to select their vector control plants from our list of 38 documented plants in Section 5 bis. In view of this contradictory result, we cannot ensure that the respondents acquired all their knowledge through ancestral transmission.

### 3.4. General Respondents’ Skills to Identify and Use Caribbean Plants

**Figure 3:**
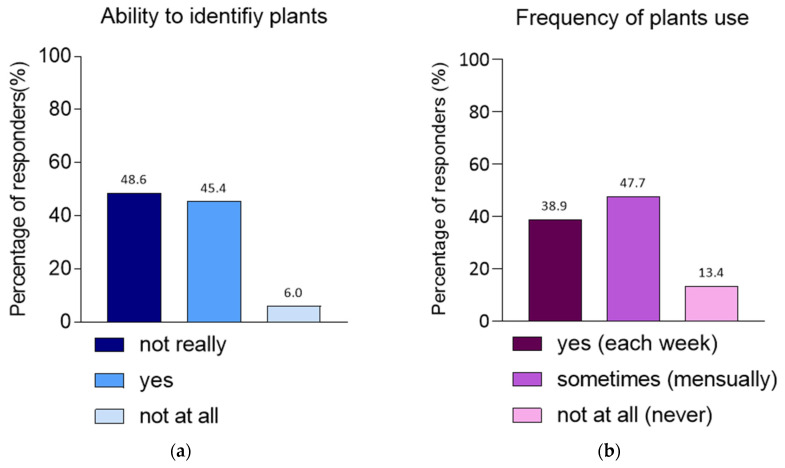

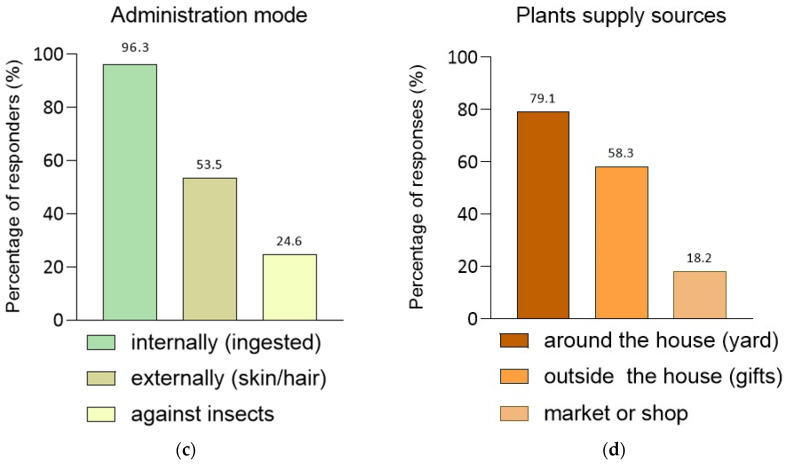
Plant identification (**a**); Frequency of plants using (**b**); Types of plants using (**c**); Plants supply locations (**d**).

All participants were asked about their general skills to identify Caribbean plants. According to the participants in the panel, 45.4% (98/216) of users could identify the plants around them, 48.6% (105/216) of informants were uncertain, and 6% (13/216) of informants were unable to identify Caribbean plants (Figure 3a). Subsequently, all participants were asked about their general skills in using Caribbean plants. The survey showed that 38.9% (84/216) of participants used plants daily, while 47.7% (103/216) of users used plants at least once a month, and 13.4% (29/216) have never used plants (Figure 3b). Internal plants use (i.e., ingestion) accounts for 96.3% (180/187) of respondent’s answers, while external use, such as application to the skin and/or hair, accounts for 53.5% (100/187) of responses; the domestic/environmental use, such as insecticide or microbial treatment, accounts for 24.6% (46/187) of responses, Figure 3c.

Finally, selected users were asked to indicate their supply locations to find or buy Caribbean plants: 79.1% (148/187) obtained their plants from a garden or a yard (around their house). Additionally, 58.3% (109/187) obtained them from their neighbours, family or friend. A smaller proportion,18.2% (34/187) purchased plants at markets, shops or Pharmacy (Figure 3b).

### 3.5. Recognizable and Mainly Used Plants

**Figure 4:**
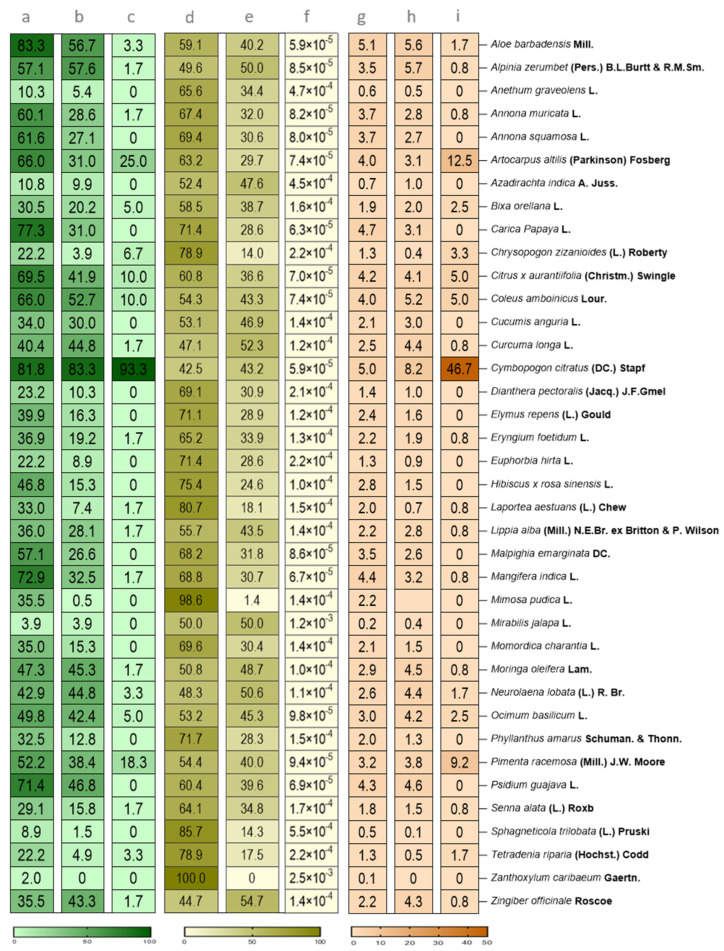
Heatmaps of the percentage of plants selected by pre-selected users for Sections bis 3, 4, and 5, along with their statistical indices: Frequency of Citation (FC) and Fidelity Level (FL). (**a**) Percentage of identified plants in Section 3 bis; (**b**) Percentage of used plants in Section 4 bis; (**c**) Percentage of plants selected for their anti-mosquito properties; (**d**) Fidelity level of selected plants in the Section 3 bis; (**e**) Fidelity level of selected plants in the Section 4 bis; (**f**) Fidelity level of selected plants in the Section 5 bis; (**g**) Frequency of citation index in Section 3 bis; (**h**) Frequency of citation index in Section 4 bis; (**i**) Frequency of citation index in Section 5 bis.

All selected users were asked to select plants throughout our 38 know-how plants proposed list. This selection allowed us to highlight the plants they can identify and use. Of 94% (203/216) of respondents who reported being able to recognize Caribbean plants, the most commonly identified plants were *Aloe barbadensis* (83.3% or 169/203), *Cymbopogon citratus* (81.8% or 166/203), *Carica papaya* (77.3% or 157/203), *Mangifera indica* (73% or 148/203) and *Annona muricata* (73% or 122/203) being the most commonly identified. However, five plants proved difficult for participants to recognize: *Zanthoxylum caribaeum* (2% or 4/203), *Mirabilis jalapa* (4% or 8/203), *Sphagneticola trilobata* (8.9% or 18/203) and *Azadirachta indica*, and *Anethum graveolens* (10.8% or 22/203 and 10.3% or 21/203 respectively). These results are presented in Figure 4, Section 3 bis. Parameters were calculated to quantify the link between recognizable plants and Guadeloupean respondents. The first set of metrics calculated is the frequency of citation, named as FC3 b in this section, and yielded across 5.05% and 0.12%. The highest index value of 5.05% was attributed to *Aloe barbadensis*, followed by *Alpinia zerumbet* at 4.97% and *Anethum graveolens* at 4.70%. The second set of metrics calculated is the index of frequency level, named as FL3 b. This index was yielded across 100% and 42.45%. Thus, the highest index value of 100% was attributed to *Aloe barbadensis*, followed by *Alpinia zerumbet* at 98.63% and *Anethum graveolens* at 85.71%. On the lower end, *Zingiber officinale* had the lowest index value of 42.45%, followed by *Zanthoxylum caribaeum* at 44.72% and *Sphagneticola trilobata* at 47.12%.

Among the 86.6% (187/216) of users who indicated frequent or regular plant usage, the most used were, *Cymbopogon citratus* (90.4% or 169/216), *Alpinia zerumbet* (65.6% or 117/216), *Aloe barbadensis* (61.5% or 115/216), *Psidium guajava* (50.8% or 95/216) and *Moringa oleifera* (49.2% or 92/216). Each plant has been reported as used by at least one respondent, except for *Zanthoxylum caribaeum*, which was not selected by any participant (0% selection or 0/216). The least frequently used plants were *Mimosa pudica* (0.5% or 1/216), *Sphagneticola trilobata* (1.6% or 3/216), *Chrysopogon zizanioides* (4.3% or 8/216), *Mirabilis jalapa* (4.3% or 8/216), and *Tetradenia riparia* (5.3% or 10/216). These results are presented in Figure 4, Section 4 bis.

Data such as the frequency of citations index (FC) and the index of level fidelity (FL) are also available, providing information on the statistical relevance of users’ responses according to the folk medicine in the different sections.

The first set of metrics in this section, named FC4b, varied between 0% and 8.18%. The highest FC4b index was 8.18%, recorded for *Aloe barbadensis*. The second highest value, 5,65%, was attributed to *Alpinia zerumbet*, *Anethum graveolens*, and *Annona muricata*. The third highest value, 4.60%, was recorded for *Annona squamosa*. Moreover, *Zingiber officinale* was the only plant with an index value of 0% in this study. Then, the second lowest index was 0.04% and was attributed to *Zanthoxylum caribaeum*. Finally, the third lowest index, 0.14%, was attributed to *Sphagneticola trilobata*. All these results were available in Figure 4, Section 4 bis.

The second set of metrics in this section index named FL4b in this section varied between 54.65% and 0%. The highest FL4b index is 54.65% and was attributed to *Aloe barbadensis*. The second highest index value is 52.29% and was attributed to *Alpinia zerumbet*. Finally, the third highest index value is 50.55% and was attributed to *Anethum graveolens*. The lowest index value (0%) was attributed to *Zingiber officinale*. The second lowest index value (1.36%) was attributed to *Zanthoxylum caribaeum*. Finally, the third lowest index value is 14.03% and was attributed to *Sphagneticola trilobata* (Figure 4, Section 4 bis).

### 3.6. Anti-Mosquito Plants Identified by the Ethnobotanical Survey

The informants who regularly use plants were surveyed about their use for vector control. Thus, 86.6% (187/216) of informants revealed that they frequently used Caribbean plants, at least once a month (Figure 3b). However, only 32% (60/187) of them use plants for their vector control properties. Out of the 38 plants listed in the survey, 22 plants were selected from the proposed panel (Table 2). The three most selected plants were *Cymbopogon citratus* (93.3% or 56/187), *Artocarpus alitis* (25.0% or 15/187), and *Pimenta racemosa* (18.3% or 11/187). On the other hand, 17 plants were not selected by any user with expertise in Caribbean plant use. These include *Anethum graveolens*, *Annona squamosa*, *Azadirachta indica*, *Carica papaya*, *Cucumis anguria*, *Dianthera pectoralis*, *Elymus repens*, *Euphorbia hirta*, *Hibiscus rosa sinensis*, *Malpighia emarginata*, *Mimosa pudica*, *Mirabilis jalapa*, *Momordica charantia*, *Phyllanthus amarus*, *Psidium guajava*, *Sphagneticola trilobata*, *and Zanthoxylum caribaeum* plants.

Different parameters were calculated to highlight the connection between plants and their various uses in folklore medicine. The first metric in this section is the frequency of citation (FC), which was calculated to assess the relevance of plant use in our study populations. Indeed, this metric indicates how often a particular plant is mentioned by informants. In the anti-mosquito section, this index, named FC5 b, ranged from 46.67% to 0%. The highest value, 46.7%, was attributed to *Aloe barbadensis*, followed by *Alpinia zerumbet* at 12.5% and *Anethum graveolens* with 9.17%. The lowest index, 0.83%, was attributed to *Cucumis anguria*, *Curcuma longa*, *Cymbopogon citratus*, *Dianthera pectoralis*, *Elymus repens*, *Eryngium foetidum*, *Euphorbia hirta*, *Hibiscus rosa sinensis*, *Lippia alba*, and *Malpighia emarginata*. The second lowest index calculated was 1.67% and was attributed to *Carica Papaya*, *Chrysopogon zizanioides*, and *Citrus aurantiifolia*. Finally, the third lowest index was 2.5% and was attributed to *Azadirachta indica* and *Bixa orellana*. Several plants were attributed to the index of 0%. These included *Coleus amboinicus*, *Laportea aestuans*, *Mangifera indica*, *Mimosa pudica*, *Mirabilis jalapa*, *Momordica charantia*, *Moringa oleifera*, *Neurolaena lobata*, *Ocimum basilicum*, *Phyllanthus amarus*, *Pimenta racemosa*, *Psidium guajava*, *Senna alata*, *Tetradenia riparia*, *Sphagneticola trilobata*, *Zanthoxylum caribaeum*, and *Zingiber officinale*.

The second set of metrics calculated is the fidelity level index (FL), which indicates the importance of specific plants in different survey sections. In the anti-mosquito plants section, the fidelity level index, named as FL5 b, ranged from 0.00250% and 0.00006%. These metrics gave very low results, with the highest value being 0.0025% for the *Zanthoxylum caribaeum* plants. The second highest value, 0.0012%, was recorded for *Mirabilis Jalapa* while the third highest value, 0.00055%, was recorded for *Sphagneticola trilobata* plants. The lowest value at 0.00006% was recorded for *Aloe barbadensis*, *Coleus amboinicus*, *Cymbopogon citratus* and *Carica papaya*. Some plants share the same FL index, such as *Mangifera indica*, *Psidium guajava*, *Citrus aurantiifolia* and *Artocarpus altilis*. Each of these plants had a value of 0.00007%. Finally, *Euphorbia hirta*, *Chrysopogon zizanioides* and *Tetradenia riparia* were indexed with a value of 0.00022%.

### 3.7. Previous Knowledge on Our Selected Anti-Mosquito Plants

The literature review conducted in this study revealed 12 plants that have not been reported for their vector control properties in the literature according to our inclusion/exclusion criteria (Table 3). These plants include *Cucumis anguria*, *Dianthera pectoralis*, *Elymus repens*, *Eryngium foetidum*, *Laportea aestuans*, *Malpighia emarginata*, *Neurolaena lobata*, *Pimenta racemosa*, *Senna alata*, *Tetradenia riparia*, *Sphagneticola trilobata* and *Zanthoxylum caribaeum*.

In contrast, 26 plants also pre-selected by our multidisciplinary groups have been studied in the literature for their vector control properties. The plant parts most commonly used for extraction are roots, stems, barks, leaves, flowers, rhizomes, latex, fruits and seeds. In this study, the most commonly used parts were the leaves, and they were extracted from the species *Aloe barbadensis*, *Annona muricata*, *Annona squamosa*, *Azadirachta indica*, *Carica papaya*, *Coleus amboinicus*, *Cymbopogon citratus*, *Euphorbia hirta*, *Lippia alba*, *Mangifera indica*, *Mimosa pudica*, *Momordica charantia*, *Moringa oleifera*, *Ocimum basilicum* and *Phyllanthus amarus*. However, the least commonly used plant part is the latex, found in *Carica papaya*.

Various extracts, including essential oils, hydrolates, aquaeous extracts, organic extracts, and powder extracts, were obtained from these plant parts using solvents like chloroform, methanol, ethanol, distilled water, and other organic solvents. Several properties of interest for vector control plants were tested on multiple mosquito stages. Larvicidal, adulticidal, pupicidal, ovicidal, as well as repellent and oviposition-deterrent properties were examined in these studies. Additionally, various mosquito species were used in these tests, including the genus *Chironomus*, *Aedes*, *Culex*, and *Anopheles*.

The experiments were carried out under different conditions, including laboratory, semi-field, field, and in vitro conditions.

## 4. Discussion

This study is the first ethnobotanical survey conducted in Guadeloupe to identify and document local plant species, particularly those with potential anti-mosquito properties.

Our interviews revealed that 94% of participants recognized Caribbean plants, and 86.6% used them regularly; only 32% employed them for mosquito control. This indicates a lack of awareness regarding their vector-control potential. *Aloe barbadensis* and *Cymbopogon citratus* were the most cited, while *Zanthoxylum caribaeum* and *Mirabilis jalapa* were less known, reflecting both the persistence of knowledge about well-known species and a gradual erosion of traditional plant use, possibly due to urbanization and shifts in cultural transmission [142,143].

The sociodemographic profile of the 216 respondents reveals that most were women (62.5%), consistent with Guadeloupe’s gender distribution [144] and ethnobotanical trends, where women are often the primary keepers of plant knowledge [145,146]. The majority were aged 18 to 35 years old (49.0%), with a notable proportion working in the health sector (27.4% of participants), suggesting a rising interest in phytotherapy among younger generations. Despite 50.8% unknown profession, responses showed diverse backgrounds, suggesting that plant knowledge is not confined to a specific profession. The low representation of agricultural workers (3.7%) may reflect limited connection with traditional practices or a sampling bias due to the online format, which may have excluded rural populations. This aligns with studies highlighting the role of land interaction in knowledge transmission, often disrupted in urban settings [147].

Regarding geographical residence, 98.1% of users lived in Guadeloupe or in the Caribbean region, reinforcing the cultural relevance of our data. A high proportion of users (76.9%) had access to a garden, mainly among those in houses, while apartment occupants had less access. This finding underscores how urban housing conditions may limit interaction with local flora. Similar patterns have been observed in other tropical regions, where reduced green space access correlates with declining ethnobotanical knowledge, particularly among urban populations [142,146].

Plant recognition was reported by 94% of respondents, with *Aloe barbadensis, Cymbopogon citratus*, and *Carica papaya*, while species such as *Zanthoxylum caribaeum* and *Mirabilis jalapa* were less familiar. This discrepancy highlights the unequal transmission of botanical knowledge and emphasizes the importance of promoting education on lesser-known plant species. *Aloe barbadensis* recorded the highest citation frequency, followed by *Alpinia zerumbet* and *Anethum graveolens. Moreover, Aloe barbadensis* recorded a perfect fidelity level of 100%, followed closely by *Alpinia zerumbet* and *Anethum graveolens*. Conversely, species like *Zingiber officinale* and *Sphagneticola trilobata* had low fidelity and citation rates. These observations are consistent with another study carried out in Guadeloupe by Courric et al. [28]. Indeed, *Aloe barbadensis* and *Alpinia zerumbet* were highly cited for identification at 12% and 18%, respectively. However, differences in the mention of species such as Mirabilis jalapa and Anethum graveolens between both studies suggest regional or generational variations in plant knowledge. This reinforces the importance of localized, regularly updated ethnobotanical surveys to capture dynamic patterns of knowledge transmission [142,146].

Regarding plant use frequency, 38.9% of participants reported daily use, while 47.7% used them at least once a month. The predominance of internal use (96.3%) over external application (53.5%) suggests widespread plant consumption, but a lower prevalence of use in dermatological or vector-related contexts. Only 24.6% of respondents claimed to use plants in domestic or environmental pest control. These results mirror the findings of Courric et al. [28], where internal uses, especially in the form of infusions, were most frequently reported.

In terms of specific use, *Cymbopogon citratus* was the most frequently used (90.4%), followed by *Alpinia zerumbet* (65.6%) and *Aloe barbadensis* (61.5%). However, plants such as *Zanthoxylum caribaeum* were not cited, which may reflect their low visibility or use in the local context. The limited usage of certain plants can also be attributed to economic factors in Guadeloupe and limited accessibility to conventional medicines. These results align with an other ethnobotanical surveys conducted in Guadeloupe, indeed, the study by Courric et al. [28], highlighted that *Cymbopogon citratus* was identified by participants as recognizable in 30% of cases among the 86 reported plant species, followed by *Aloe vera* (19%) and *Alpinia zerumbet* (approximately 12%), reflecting a stronger familiarity with these species within the Guadeloupean population.

The high fidelity of *Aloe barbadensis* (54.65%), *Alpinia zerumbet* (52.29%), and *Anethum graveolens* (50.55%) affirms their medicinal relevance in local practices. Conversely, *Zingiber officinale* (0%) and *Zanthoxylum caribaeum* were rarely cited or selected, indicating low visibility or limited perceived usefulness. Despite *Aloe barbadensis* having the highest citation frequency (8.18%), its fidelity index for vector control use (FL5b) was only 0.00006%, reflecting a significant mismatch between recognition and targeted application. Overall, while 86.6% of participants reported using medicinal plants, only 32% used them for mosquito control, highlighting a substantial gap in public awareness of their full bioactive potential.

Out of the 38 proposed plants, only 22 were selected, with Cymbopogon citratus cited by 93.3% of respondents, followed by *Pimenta racemosa* (18.3%). The exclusion of 17 species suggests selective cultural salience, a common trend in ethnobotany, where a few emblematic plants dominate collective knowledge and use. These findings underscore the importance of developing educational strategies, particularly for younger generations and urban populations with limited green space access, to bridge the gap between traditional plant knowledge and its application in sustainable, culturally relevant vector control strategies [143,147,148,149].

Regarding participants’ skills, our pre-selection method revealed that 78.2% of the total participants were not assessed on their vector control plant skills because of their inability to identify or list their usages for surrounding plants. This result is alarming because only 21.2% of participants were able to correctly identify plants. This lack of evaluation could indicate that the transmission of ancestral knowledge about plants is not systematic among respondents. This finding suggests that a large proportion of the population may not be sufficiently informed about several cultural practices related to local plants as vector control properties. This raises serious concerns about the continuity of ethnobotanical knowledge transmission, especially in the context of urbanization and reduced contact with nature [142,146].

To refine our analysis, we conducted a comparative literature review based on inclusion/exclusion criteria. Our findings revealed that 12 plants reported by our respondents had no prior documentation regarding vector control properties, including *Cucumis anguria*, *Dianthera pectoralis*, *Elymus repens*, *Eryngium foetidum*, *Laportea aestuans*, *Malpighia emarginata*, *Neurolaena lobata*, *Pimenta racemosa*, *Senna alata*, *Tetradenia riparia*, *Sphagneticola trilobata*, and *Zanthoxylum caribaeum*. This underlines their potential as underexplored bioresources and highlights the need to expand the scope of pharmacological screenings beyond the usual targets.

Among these species, the most frequently cited by respondents for their potential vector control properties were *Artocarpus altilis* (25%), *Pimenta racemosa* (18.3%), *Bixa orellana* (5%), *Tetradenia riparia* (3.3%), as well as *Eryngium foetidum*, *Laportea aestuans*, and *Senna alata* (each 1.7%). However, the case of *Pimenta racemosa* is particularly compelling, while no English-language studies met our inclusion criteria for the literature review, research conducted in Cuba by Leyva et al. [150] and published in Spanish, documented its potential larvicidal and repellent activity against *Aedes aegypti*. Our work thus echoes recent calls in ethnobotany and ecology to integrate multilingual and grey literature into scientific syntheses to avoid bias and knowledge exclusion [151]. Moreover, previous research by Abaul et al. [152] revealed the existence of three chemotypes of *Pimenta racemosa*, whose potential vector control properties have not yet been studied, suggesting that further investigations are needed to determine whether each chemotype exhibits vector control properties.

Interestingly, some plants that are well-documented in the scientific literature for their vector control properties, were not selected by our pre-selected respondents such as *Annona squamosa* L. reported for adulticidal and larvicidal activity against mosquitoes [65,66]; *Azadirachta indica* reported for their multiple properties against mosquitoes [78,82,153]; *Carica papaya* reported for larvicidal and attractive activity [83,89]; *Euphorbia hirta* reported for their multiple properties against [108,110]; *Hibiscus rosa sinensis* reported for larvicidal properties [111]; *Mimosa pudica* reported for their larvicidal, adulticidal and repellent activity; *Mirabilis jalapa* reported for larvicidal activity [118]; *Momordica charantia* reported for larvicidal [64,111,120,121]; *Phyllanthus amarus* reported for larvicidal and repellent test [68,69] and *Psidium guajava* reported for their multiple properties against mosquitoes [121,134,135]. This may reflect a disconnection between scientific literature and popular use, influenced by accessibility to extraction technologies, cultural familiarity, or even the perceived relevance of plants for vector control [142,154].

Among these twelve well-documented plants, several share common molecular families, raising the question of whether their vector control properties could be attributed to specific chemical compounds. Identifying these shared bioactive molecule families could help validate their role in vector control and provide insights into potential synergistic mechanisms.

Additionally, our analysis revealed that certain botanical families are particularly well-represented among plants with reported vector control properties, including Zingiberaceae, Poaceae, and the Lamiaceae family, each of which contains multiple species identified for their potential vector control effects. The recurrence of these families across active species raises the possibility that phytochemical activities may be family-specific, providing a useful taxonomic filter for future screening and drug discovery [149].

Furthermore, an in-depth examination of the twelve well-documented plants revealed that several phytochemical families appear repeatedly across different species. Notably, tannins are found in *Azadirachta indica*, *Carica papaya*, *Mimosa pudica*, *and Phylanthus amarus*. Flavonoids are present in *Annona squamosa*, *Euphorbia hirta*, *Hibiscus rosa-sinensis*, *Momordica charantia*, *Phylanthus amarus*, and *Psidium guajava*. Triterpenes are reported in *Euphorbia hirta*, *Momordica charantia*, *Phylanthus amarus*, and *Psidium guajava*, while phenolic compounds are found in *Annona squamosa*, *Carica papaya*, *Momordica charantia*. These recurrent phytochemical families are known for their insecticidal, larvicidal, and repellent properties, and may act through synergistic mechanisms targeting mosquito physiology or behavior.

Limiting factors: To strengthen this study, it would have been beneficial to clarify the broader context of plant-based vector control efficacy in the survey. Participants should have been informed that any effect on mosquito behavior (whether repellent, lethal, or influencing interactions after a bite) was relevant, helping to expand the focus beyond lethal effects alone.

The absence of photographs of the plants included in our questionnaire is a significant limitation, as it likely hindered the ability of respondents to recognize certain species. Indeed, visual cues are critical in ethnobotanical identification and can bridge gaps between linguistic and sensory recognition [155].

Moreover, the language restriction applied in the literature review methodology inevitably led to the exclusion of relevant studies published in other languages, particularly Spanish, which are predominant in the tropical regions where mosquito-borne diseases are also prevalent, as Cuba, where arbovirus transmissions such as dengue virus, chikungunya, and Zika have been previously [156,157].

Additionally, the reliance on self-reported data in our ethnobotanical survey introduces the possibility of declarative bias. Respondents’ responses may be influenced by personal perceptions, memory recall, or varying levels of botanical knowledge. Moreover, the restrictions imposed by the COVID-19 pandemic required that the survey be conducted exclusively online, using a digital questionnaire [30]. While this approach ensured accessibility and data collection efficiency, it limited the possibility of obtaining testimonies from elders with extensive knowledge of ancestral practices. This may have resulted in an underrepresentation of certain traditional uses that are less known among younger generations. However, efforts were made to optimize the questionnaire design by ensuring that the questions were short, simple, yet precise, reducing the completion time to approximately five minutes and minimizing respondent fatigue.

Finally, broader environmental and sociocultural factors must be considered. The link between urbanization and plant knowledge transmission is a crucial aspect, as limited access to green spaces in apartment settings may reduce direct exposure to local flora and contribute to a gradual loss of traditional knowledge. This could explain the lower recognition rates of certain lesser-known species in our survey. Additionally, as knowledge about medicinal plants is often passed down through generations, shifting cultural practices and modernization may further impact the transmission and preservation of ethnobotanical traditions in Guadeloupe and other Caribbean regions.

## 5. Conclusions

Caribbean plants commonly used against the *Aedes aegypti* mosquito have great potential for mosquito control. However, knowledge about these plants in Guadeloupe remains poorly documented in the literature. The results of this first ethnobotanical study focusing on vector control plants highlight the potential of 22 species recognized by the surveyed participants for their anti-mosquito properties. Notably, *Cymbopogon citratus*, *Pimenta racemosa* (including its sub-varieties), *Artocarpus altilis*, and *Tetradenia riparia* emerged as the most frequently cited plants for mosquito control. In contrast, other plants with vector control properties well-documented in literature, such as *Azadirachta indica* and *Carica papaya*, were not selected by the participants. This discrepancy underscores a significant gap between scientific knowledge and traditional or cultural perceptions of plant efficacy, emphasizing the need for further interdisciplinary research to bridge this divide and integrate local knowledge into scientific validation processes.

A comparative analysis with existing literature identified 12 plants not previously reported for vector control properties, suggesting new research opportunities for their potential larvicidal, adulticidal, or repellent effects, as well as highlighting the value of ethnobotanical surveys in uncovering underexplored plant-based solutions. Although low citation frequencies and fidelity indexes were obtained in some cases, they provide valuable insight into the cultural significance of certain plants in local practices for internal, external, and environmental usages.

These findings present promising opportunities for further scientific investigation into Caribbean plants, particularly as eco-friendly and sustainable alternatives for mosquito control. They also emphasize the importance of preserving and transmitting traditional knowledge on plant usage, while raising awareness of their potential in vector management strategies. Finally, the identification of lesser-studied yet promising plants calls for additional studies, in laboratory and field studies to assess their effectiveness under controlled and real-world conditions. In a context where mosquito resistance to synthetic insecticides is increasing, plant-based approaches offer promising alternatives to enhance vector control strategies in Guadeloupe and beyond.

## Figures and Tables

**Table 2:** Age distribution of respondents across work sectors by age group.

Work Sector	18–35 Years Old	36–50 Years Old	51–65 Years Old	66 Years and More	Total
Other sector	52.8% (56/106)	41.4% (12/29)	44.0% (22/50)	61.3% (19/31)	50.5% (109/216)
Farming sector	0.9% (1/106)	6.9% (2/29)	6.0% (3/50)	6.4% (2/31)	3.7% (8/216)
Education sector	4.7% (5/106)	6.9% (2/29)	32.0% (16/50)	9.7% (3/31)	12.0% (26/216)
Research sector	14.2% (15/106)	24.1% (7/29)	8.0% (4/50)	9.7% (3/31)	13.4% (29/216)

**Table 3:** Comparison of vector-control properties from literature and ethnobotanical survey: study based on the 38 know-how plants list.

Scientifics Names Plants (Family)	Anti-Mosquito Plants Reported in This Study (%)	Plants Parts Reported in the Litterature	Type of Extracts Used	Target Mosquitoes Species	Bioassays Performed	Experimental Condition: Field (F), Laboratory (L), or In Vitro (V)	References
*Aloe barbadensis* Mill. (Asphodelaceae)	3.3	Leaves	Powder	*Aedes aegypti*	Larvicidal	L	[62]
*Alpinia zerumbet* (Pers.) B.L.Burtt & R.M.Sm. (Zingiberaceae)	1.7	Flowers	Essential oil	*Aedes aegypti*	Repellent, Irritant, Toxicity	L	[63]
*Anethum graveolens* L. (Apiaceae)	0	-	-	*-*	-	-	-
*Annona muricata* L. (Annonaceae)	1.7	Seeds, Leaves, Stems	Aqueous, Oils, Ethanolic	*Culex quiquefaciatus*, *Aedes albopictus* and *Aedes aegypti*	Adulticidal, Larvicidal	L	[64,65]
*Annona squamosa* L. (Annonaceae)	0	Seeds, Leaves, Stems, Bark, Root Bark	Aqueous, Oils, Organics, Methanolic	*Culex quiquefaciatus*, *Aedes albopictus*, *Aedes aegypti*, *Anopheles stephensi*, *Culex tritaeniorrhynchus* and *Anopheles gambiae*	Adulticidal, Larvicidal	L	[64,65,66,67,68,69,70]
*Artocarpus altilis* (Parkinson) Fosberg (Moraceae)	25	-	-	*-*	-	-	-
*Azadirachta indica* A. Juss. (Meliaceae)	0	Seeds, Leaves, Neem cake, Seed, Fruits and Bark	Organics, commercial preparation, Powder, Smoked leaves, Commercial oil, Oil cream, Emulsified neem oil, Aqueous, Crude extract of leaves and powders, Essential oils	*Anopheles aquasalis*, *Anopheles gambiae*, *Anopheles stephensi*, *Culex quinquefasciatus*, *Anopheles arabiensis*, *Aedes aegypti*, *Anopheles culicifacies, Anopheles stephensi*, *Chironomus circumdatus*, *Anopheles arabiensi*, *Anopheles gambiae*, *Aedes aegypti*, *Aedes family* and *Anopheles coluzzii*	Blood-feeding, Repellent test, Larvicidal test, Survival test (larvae), Ovicidal test, Adulticidal test, Oviposition test, Pupicidal test, Attract and kill test	L, F and V	[19,71,72,73,74,75,76,77,78,79,80,81,82,83,84,85,86]
*Bixa orellana* L. (Bixaceae)	5	-	-	*-*	-	-	-
*Carica papaya* L. (Caricaceae)	0	Seeds, Leaves, Fruit flesh and peels, Granules, Stems, Latex	Powder, Ethanolic, Organic and Essential oil	*Aedes aegypti*, *Aedes albopictus* and *Culex quinquefasciatus*	Larvicidal test, Mosquito attractive test	L	[64,83,87,88,89,90]
*Chrysopogon zizanioides* (L.) Roberty (Poaceae)	6.7	ND	Nanoemulsions of essential oil	*Aedes aegypti* and *Anopheles minimus*	Repellent test, Mosquito Repellent, Efficiency assay	L	[91,92]
*Citrus* × *aurantiifolia* (Christm.) Swingle (Rutaceae)	10	-	-	*-*	-	-	-
*Coleus amboinicus* Lour. (Lamiaceae)	10	Leaves	Essential oil	*Anopheles gambiae*	Latvicidal test, Adulticidal test	L	[93]
*Cucumis anguria* L. (Cucurbitaceae)	0	-	-	*-*	-	-	-
*Curcuma longa* L. (Zingiberaceae)	1.7	Tuber, Rhizomes	Crude and chloroform: methanol (1:1), Essential oil, Organic, Formulation (7 plants)	*Culex pipiens, Anopheles stephensi*, *Culex quinquefasciatus*, *Aedes albopictus* and *Aedes aegypti*		L and F	[94,95,96,97,98]
*Cymbopogon citratus* (DC.) Stapf (Poaceae)	93.3	Leaves, Stems, Roots, Whole plants	Aqueous, Ethanolic, Essential oil	*Culex quinquefasciatus*, *Aedes aegypti*, *Anopheles gambiae*, *Anopheles funestus*, *Aedes aegypti*, *Aedes albopictus*, *Ae. aegypti Anopheles dirus*, *Culex quinquefasciatus* and *Anopheles darlingi*	Larvicidal test, Repellent test, Adulticidal test, Ovicidal test, Pupicidal test, Oviposition-deterent test	F	[99,100,101,102,103,104,105,106,107]
*Dianthera pectoralis* (Jacq.) J.F.Gmel. (Acanthaceae)	0	-	-	*-*	-	-	-
*Elymus repens* (L.) Gould (Poaceae)	0	-	-	*-*	-	-	-
*Eryngium foetidum* L. (Apiaceae)	1.7	-	-	*-*	-	-	-
*Euphorbia hirta* L. (Euphorbiaceae)	0	Leaves	Organic solvent	*Anopheles stephensi*	Larvicidal test, Puppicidal test, Adulticidal test, Adult emergence inhibition test, Ovicidal test, Repellent test	L	[108,109,110]
*Hibiscus* × *rosa-sinensis* L. (Malvaceae)	0	Flowers	Organic extract	*Culex quinquefasciatus*	Larvicidal test	L	[111]
*Laportea aestuans* (L.) Chew (Urticaceae)	1.7	-	-	*-*	-	-	-
*Lippia alba* (Mill.) N.E.Br. ex Britton & P.Wilson (Verbenaceae)	1.7	Plant material, Leaves	Essential oil	*Aedes aegypti*, *Aedes aegypti*, *Culex quinquefasciatus* larvae, *Anopheles gambiae* and *Aedes aegypti*	Larvicidal test, Adulticidal test, Repellent test, Pupicidal test, Oviposition-deterent	L	[103,112,113,114]
*Malpighia emarginata* DC. (Malpighiaceae)	0	-	-	*-*	-	-	-
*Mangifera indica* L. (Anacardiaceae)	1.7	Stems, Peels, Leaves and Bark	ND	*Aedes aegypti*, *Aedes albopictus*, *Anopheles stephensi* and *Culex quinquefasciatus*	Larvicidal test	L	[54,115,116]
*Mimosa pudica* L. (Fabaceae)	0	Leaves	Organic extract	*Culex gelidus Theobald* and *Culex quinquefasciatus*	Larvicidal test, Adulticidal test, Repellent test		[117]
*Mirabilis jalapa* L. (Nyctaginaceae)	0	Leaves	Organic extract	*Culex quinquefasciatus*, *Aedes aegypti* and *Anopheles stephensi*	Larvicidal test	L	[118]
*Momordica charantia* L. (Cucurbitaceae)	0	Leaves, Stems, flowers, Fruits, Fresh leaves	Organic, Crude, Essential oil	*Aedes aegypti*, *Culex gelidus*, *Culex quinquefasciatus*, *Anopheles stephensi*	Larvicidal test	L	[64,119,120,121]
*Moringa oleifera* Lam. (Moringaceae)	1.7	Seeds, Leaves	Powder, Aquaeous, Methanolic, Essential oil, Water extract of Moringa oleifera seeds (WEMOS)	*Aedes aegypti*, *Anopheles stephensi* and *Anopheles gambiae*	Larvicidal test, Ovicidal test, Oviposition test, Pupicidal test, Egg Hatching test, Repellent test, Forearm attraction test	L	[122,123,124,125,126]
*Neurolaena lobata* (L.) R. Br. ex Cass. (Asteraceae)	3.3	-	-	*-*	-	-	-
*Ocimum basilicum* L. (Lamiaceae)	5	Leaves, Aerial parts	Essential oil	*Culex pipiens*, *Anopheles stephensi*, *Aedes aegypti* and *Anopheles gambiae*	Larvicidal test, Adulticidal test, Adults emergence inhibition test, Repellent test, Forearm test	L	[110,123,127,128,129,130]
*Phyllanthus amarus* Schumach. & Thonn. (Phyllanthaceae)	0	Leaves and stem of P. amarus	Organic	*Anopheles stephensi*, *Aedes aegypti*, *Culex tritaeniorhynchus*, and *Culex quinquefasciatus*	Larvicidal test, Repellent test	L	[68,69]
*Pimenta racemosa* (Mill.) J. W. Moore (Myrtaceae)	18.3	-	-	*-*	-	-	-
*Psidium guajava* L. (Myrtaceae)	0	Plant materials, Leaves, Fruits, Fresh leaves, Guava,	crude dried residues, ethanolic, ash, essential oil, nectar, fruit solution	*Aedes aegypti*, *Anopheles minimus*, *Anopheles epiroticus*, *Culex. Quinquefasciatus*, *Anopheles arabiensis*, *Aedes albopictus*, *Culex fuscocephala*, *Anopheles stephensi, Anopheles gambiae, Culex* spp. and *Anopheles* spp.	Larvicidal test, Adulticidal test, Repellent test, Free-flight attraction assays, Attract and kill test	L	[121,131,132,133,134,135,136]
*Senna alata* (L.) Roxb. (Fabaceae)	1.7	-	-	*-*	-	-	-
*Sphagneticola trilobata* (L.) Pruski (Asteraceae)	0	-	-	*-*	-	-	-
*Tetradenia riparia* (Hochst.) Codd (Lamiaceae)	3.3	-	-	*-*	-	-	-
*Zanthoxylum* caribaeum Gaertn. (Rutaceae)	0	-	-	*-*	-	-	-
*Zingiber officinale* Roscoe (Zingiberaceae)	1.7	Rhizome, Roots, Fresh samples, Fresh rhizomes	Essential oils, Formulations (7 plants), Organic	*Culex tritaeniorhynchus*, *Anopheles subpictus*, *Culex pipiens*, *Aedes aegypti*, *Anopheles funestus*, *Anopheles gambiae*, *Anopheles pharoensis*, *Culex antennatus*, *Culex quinquefasciatus*, *Culex theileri* and *Aedes albopictus*	Larvicidal test, Repellent test, Adultiticidal test, Ovicidal test, Identification of vectors test, Olfactometry-bioassys test	L	[94,101,137,138,139,140,141]

## Data Availability

The datasets generated during and/or analyzed during the current study are available from the corresponding author on reasonable request.

## Supplementary Materials

The following supporting information can be downloaded at: https://www.mdpi.com/article/10.3390/biology14070888/s1, Figure S1: The five most selected plants for their anti-mosquito properties by the panel in the survey; Figure S2: Ethnobotanical survey presented in our study.

## Abbreviations

The following abbreviations are used in this manuscript:

DENV	Dengue virus
FC	Frequency of Citation
FL	Fidelity Level
VCP	Vector control plants
VOCs	Volatile organic compound
TRAMIL	Program of Applied Research on Popular Medicine in the Caribbean
