# Supplementary material for: Contrasted Ethnobotanical and Literature Knowledge of Anti-Mosquito Plants from Guadeloupe
Source: Biology (Basel). 2025 Jul 19;14(7):888. doi: 10.3390/biology14070888 (PMC12292490; doi:10.3390/biology14070888)
Supplement: Supplementary file 1 [file biology-14-00888-s001.zip › biology-3701137-supplementary.pdf]

Supplementary data for manuscript: Contrasted Ethnobotanical and Literature Knowledge of Anti-Mosquito Plants from Guadeloupe

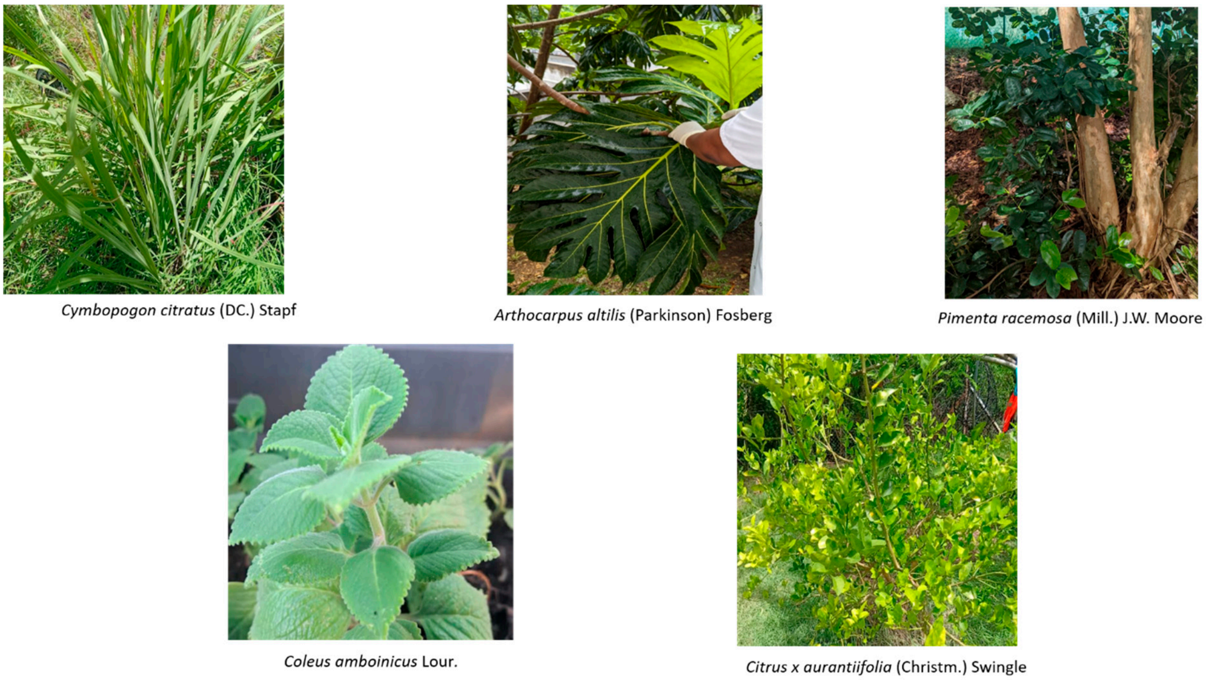

Figure S1. The five most selected plants for their anti-mosquito properties by the panel in the survey

Survey Questionnaire

Section 1 : Description of the socio demographic characteristics of users

1) What is your age group?

a) 0 – 17 years
b) 18 – 35 years
c) 36 – 50 years
d) 51 – 65 years
e) 65 years and older

2) Do you live in Guadeloupe or the Caribbean?

a) In Guadeloupe
b) In the Caribbean

3) What is your professional field?

a) Education
b) Medical
c) Research
d) Agriculture
e) Other

Section 2 : Description of the respondent's environments

4) Do you live in:

a) An apartment
b) A house

5) Do you have access to a garden?

a) Yes
b) No

6) Where do you usually obtain the plants you use? (Multiple answers allowed)

a) Around the house: yard or garden
b) Outside the home: neighbors, family, friends
c) Market or store
d) Pharmacy
e) Other

Section 3 : Plant identification skills

7) Can you identify the plants around you?

a) Yes
b) Not really
c) Not at all

Section 3 bis) Select the plants, trees, or plant parts present in your garden (or nearby):

- *Aloe barbadensis Mill.*
- *Alpinia zerumbet*
- *Anethum graveolens L.*
- *Annona muricata*
- *Annona squamosa*
- *Artocarpus altilis*
- *Azadirachta indica*
- *Bixa orellana L.*
- *Carica papaya*

- *Chrysopogon zizanioides*
- *Citrus x aurantiifolia*
- *Coleus amboinicus*
- *Cucumis anguria*
- *Curcuma longa*
- *Cymbopogon citratus*
- *Dianthera pectoralis*
- *Elymus repens*
- *Eryngium foetidum*
- *Euphorbia hirta*
- *Hibiscus rosa-sinensis*
- *Laportea aestuans*
- *Lippia alba*
- *Malpighia emarginata*
- *Mangifera indica*
- *Mimosa pudica*
- *Mirabilis jalapa*
- *Momordica charantia*
- *Moringa oleifera*
- *Neurolaena lobata*
- *Ocimum basilicum*
- *Phyllanthus amarus*
- *Pimenta racemosa*
- *Plectranthus amboinicus*
- *Psidium guajava*
- *Senna alata*
- *Sphagneticola trilobata*
- *Tetradenia riparia*
- *Zanthoxylum caribaeum*
- *Zingiber officinale*

Section 4 : Plant usage skills

8) Do you usually use medicinal plants?

a) Yes
b) No

9) How Do you use plants ?

- Internally : ingested (infusions, decoctions, macerations)
- Externally : applied to skin and hair
- For the home/environment : treatment against insects or microbes

Section 4 bis) If yes, which ones?

- *Aloe barbadensis Mill.*
- *Alpinia zerumbet*
- *Anethum graveolens L.*
- *Annona muricata*
- *Annona squamosa*
- *Artocarpus altilis*
- *Azadirachta indica*
- *Bixa orellana L.*
- *Carica papaya*
- *Chrysopogon zizanioides*
- *Citrus x aurantiifolia*
- *Coleus amboinicus*
- *Cucumis anguria*
- *Curcuma longa*
- *Cymbopogon citratus*
- *Dianthera pectoralis*
- *Elymus repens*
- *Eryngium foetidum*
- *Euphorbia hirta*
- *Hibiscus rosa-sinensis*
- *Laportea aestuans*
- *Lippia alba*
- *Malpighia emarginata*
- *Mangifera indica*
- *Mimosa pudica*
- *Mirabilis jalapa*
- *Momordica charantia*
- *Moringa oleifera*
- *Neurolaena lobata*
- *Ocimum basilicum*
- *Phyllanthus amarus*
- *Pimenta racemosa*
- *Plectranthus amboinicus*
- *Psidium guajava*
- *Senna alata*
- *Sphagneticola trilobata*
- *Tetradenia riparia*
- *Zanthoxylum caribaeum*
- *Zingiber officinale*

Section 5 : Vector control plant (VCP) skills

10) Do you use plants or tree parts externally to repel mosquitoes?

a) Yes
b) No

Section 5 bis) If yes, which ones ?

- *Aloe barbadensis Mill.*
- *Alpinia zerumbet*
- *Anethum graveolens L.*
- *Annona muricata*
- *Annona squamosa*
- *Artocarpus altilis*
- *Azadirachta indica*
- *Bixa orellana L.*
- *Carica papaya*
- *Chrysopogon zizanioides*
- *Citrus x aurantiifolia*
- *Coleus amboinicus*
- *Cucumis anguria*
- *Curcuma longa*
- *Cymbopogon citratus*
- *Dianthera pectoralis*
- *Elymus repens*
- *Eryngium foetidum*
- *Euphorbia hirta*
- *Hibiscus rosa-sinensis*
- *Laportea aestuans*
- *Lippia alba*
- *Malpighia emarginata*
- *Mangifera indica*
- *Mimosa pudica*
- *Mirabilis jalapa*
- *Momordica charantia*
- *Moringa oleifera*
- *Neurolaena lobata*
- *Ocimum basilicum*
- *Phyllanthus amarus*
- *Pimenta racemosa*
- *Plectranthus amboinicus*
- *Psidium guajava*
- *Senna alata*
- *Sphagneticola trilobata*
- *Tetradenia riparia*
- *Zanthoxylum caribaeum*
- *Zingiber officinale*

Figure S2. Ethnobotanical survey presented in our study.
